# Design, Synthesis and Antimicrobial Evaluation of New *N*-(1-Hydroxy-1,3-dihydrobenzo[*c*][1,2]oxaborol-6-yl)(hetero)aryl-2-carboxamides as Potential Inhibitors of Mycobacterial Leucyl-tRNA Synthetase

**DOI:** 10.3390/ijms24032951

**Published:** 2023-02-02

**Authors:** Petr Šlechta, Adam Anthony Needle, Ondřej Jand’ourek, Pavla Paterová, Klára Konečná, Pavel Bárta, Jiří Kuneš, Vladimír Kubíček, Martin Doležal, Marta Kučerová-Chlupáčová

**Affiliations:** 1 Department of Pharmaceutical Chemistry and Pharmaceutical Analysis, Faculty of Pharmacy in Hradec Králové, Charles University, 500 05 Hradec Králové, Czech Republic; 2 Department of Biological and Medical Sciences, Faculty of Pharmacy in Hradec Králové, Charles University, 500 05 Hradec Králové, Czech Republic; 3 Department of Clinical Microbiology, University Hospital, 500 05 Hradec Králové, Czech Republic; 4 Department of Biophysics and Physical Chemistry, Faculty of Pharmacy in Hradec Králové, Charles University, 500 05 Hradec Králové, Czech Republic; 5 Department of Organic and Bioorganic Chemistry, Faculty of Pharmacy in Hradec Králové, Charles University, 500 05 Hradec Králové, Czech Republic

**Keywords:** antimicrobial, antimycobacterial, benzoxaborole, cytotoxicity, molecular docking, multidrug-resistant tuberculosis, tuberculosis

## Abstract

Tuberculosis remains a serious killer among infectious diseases due to its incidence, mortality, and occurrence of resistant mycobacterial strains. The challenge to discover new antimycobacterial agents forced us to prepare a series of *N*-(1-hydroxy-1,3-dihydrobenzo[*c*][1,2]oxaborol-6-yl)(hetero)aryl-2-carboxamides 1–19 via the acylation of 6-aminobenzo[*c*][1,2]oxaborol-1(3*H*)-ol with various activated (hetero)arylcarboxylic acids. These novel compounds have been tested in vitro against a panel of clinically important fungi and bacteria, including mycobacteria. Some of the compounds inhibited the growth of mycobacteria in the range of micromolar concentrations and retained this activity also against multidrug-resistant clinical isolates. Half the maximal inhibitory concentrations against the HepG2 cell line indicated an acceptable toxicological profile. No growth inhibition of other bacteria and fungi demonstrated selectivity of the compounds against mycobacteria. The structure–activity relationships have been derived and supported with a molecular docking study, which confirmed a selectivity toward the potential target leucyl-tRNA synthetase without an impact on the human enzyme. The presented compounds can become important materials in antimycobacterial research.

## 1. Introduction

**Figure 1:**
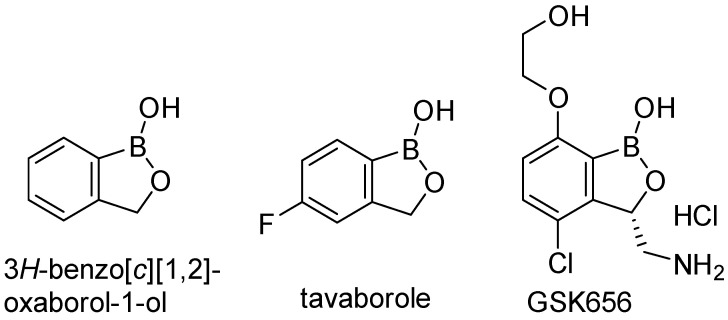
3*H*-benzo[*c*][1,2]oxaborol-1-ol and its important derivatives with antimicrobial activity.

Tuberculosis (TB), a life-threatening infectious disease, is one of the leading causes of death around the world. TB was ranked as the second cause of death from a single infectious agent in 2021 and even surpassed HIV. TB is caused by the bacillus *Mycobacterium tuberculosis* (*Mtb*), which is spread via droplets from infected people. An estimated 10.6 million people fell ill in 2021 with TB, which is a slight increase from 10.1 million in 2020 [1]. The current treatment for TB requires 6–9 months of long combinational therapy consisting of four first-line drugs such as rifampicin, isoniazid, pyrazinamide, and ethambutol. Unfortunately, emerging drug resistance reveals drug-resistant TB strains that continue to be a health threat; multidrug-resistant TB (MDR-TB) [2] and extensively drug-resistant TB (XDR-TB) [3] are especially causing most of the first-line and second-line anti-TB drugs to be ineffective. This creates an urgent need for the development of new antimycobacterial agents with a unique mechanism of action.

(3*H*)-Benzo[*c*][1,2]oxaborol-1-ol (Figure 1, often abbreviated in publications as benzoxaborole) is a derivative of boronic acid combining a cyclic hemiester moiety with a free hydroxyl group. Boron in the molecule represents a strong Lewis acidic center and the benzoxaboroles are considered more acidic than arylboronic acids [4]. Benzoxaboroles show chemical stability and high hydrolytic resistance of the boron–carbon bond. Boron-containing compounds are able either to create an ester bond or a dative bonding to the active site of enzymes [5]. Synthesis of the unsubstituted (3*H*)-benzo[*c*][1,2]oxaborol-1-ol was published in 1957 [6] and it was originally called boronophthalide [7]. Although it was synthesized 65 years ago, it had not been employed in drug design and discovery until the last two decades. The biological activities of benzoxaboroles have been reviewed in several publications [4,8,9,10]. Tavaborole (Figure 1) is the first clinically used drug in the field of antimicrobial therapy approved for the treatment of onychomycoses by the US Food and Drug Administration in 2014 [11]. The mechanism of action is the inhibition of leucyl-tRNA synthetase (LeuRS) via the oxaborole tRNA-trapping (OBORT) mechanism. The OBORT mechanism utilizes the ability of the boron atom to bond to the *cis*-diols of the 3’-terminal adenosine nucleotide Ade76 of tRNA^Leu^. The resulting covalent adduct traps the 3’ end of tRNA^Leu^ in the editing site of LeuRS and creates a nonproductive complex. This causes inhibition of leucylation and, thus, the inhibition of protein synthesis [12]. In recent years, a series of 3-aminomethylbenzoxaboroles targeting *Mtb* LeuRS by the OBORT mechanism has been identified. Thorough research focused on these derivatives led to detailed structure–activity relationship (SAR) studies and the identification of a potent *Mtb* LeuRS inhibitor with oral bioavailability and in vivo efficacy, with the 3-aminomethylbenzoxaborole derivative GSK656 (Figure 1, IC_50_ against LeuRS = 0.20 μM) submitted into clinical trials [13,14], currently in Phase 2 [15]. However, the majority of the candidates struggled with potential toxicity issues caused by the inhibition of mammalian cytoplasmic LeuRS [13]. In the work of Hu and colleagues [16], a distinctive structural difference between the editing domain of bacterial (*Streptococcus pneumoniae*) and human cytosolic LeuRS was mentioned as well as the fact that the human cytosolic LeuRS editing domain is more compact due to the presence of four additional eukaryote-specific insertion [17]. One of these additional insertions covers the opening of the editing pocket and causes the binding of more bulky compounds to be difficult [13]. We expected that a similar difference could be found between the editing domain of mycobacterial LeuRS and human cytosolic LeuRS and should serve as valuable information for the design of new selective derivatives. Different biological targets have been also described for benzoxaboroles in bacteria and fungi, e.g., β-lactamases, D,D-carboxypeptidase [4], or NADH dehydrogenase [18]; however, LeuRS is the most often mentioned and is probably the only well-described target in mycobacteria [8]. Benzoxaborole-derived compounds with excellent antimycobacterial activity [19,20,21,22] and concurrently LeuRS inhibitory activity [23,24,25,26] have been published. Many compounds have been patented and some of them reached clinical trials. Benzoxaboroles might be also suitable for surface functionalization modification or employment into co-polymers with PEG or polyacrylamide [4]. General inhibitors of microbial LeuRS have been summarized in a review article by Zhang and Ma in 2019 [27].

**Figure 2:**
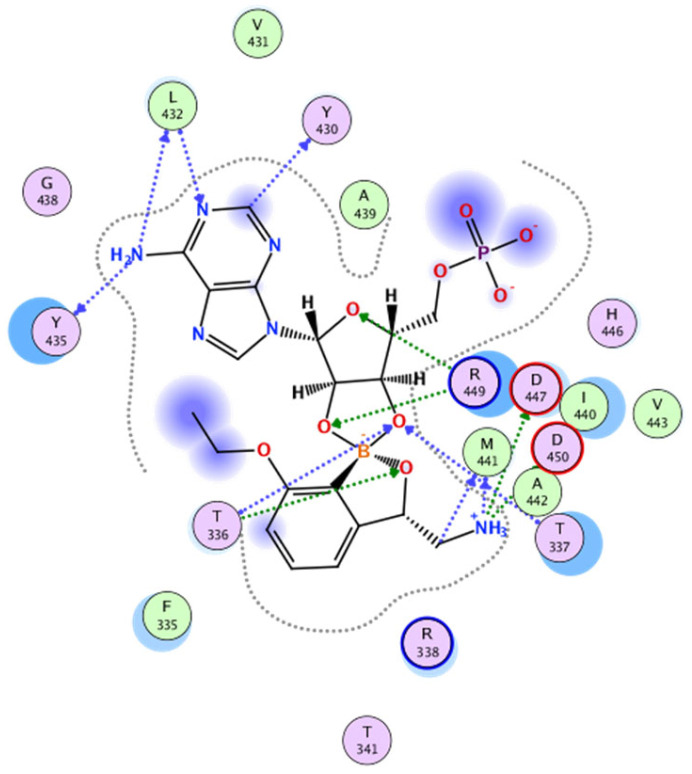
Ligand interaction diagram of *Mtb* H37Rv LeuRS and (*S*)-3-(aminomethyl)-7-ethoxybenzo[c][1,2]-oxaborol-1(*3H*)-ol in the form of the bivalent adduct with AMP (PDB ID: 5AGR).

The design of our compounds started with the experience of the above-mentioned research group [13]. The bidentate covalent adduct of benzoxaborole with Ade76 of tRNA may be mimicked by an adduct with adenosine monophosphate (AMP) [28,29]. Under the proper inspection of the crystallographic structure of *Mtb* H37Rv LeuRS co-crystallized with a confirmed inhibitor in the form of the spiro adduct with AMP (PDB ID: 5AGR, see Figure 2), it is visible that most of the H-bond interactions occur between the merged ribose part of the adduct and amino acid residues T336 and T337 of the threonine-rich region. There are several other H-bond interactions and hydrophobic contacts between L432 and Y435 of the AMP binding loop and AMP. Finally, the research group described three additional key interactions of the amino group of (*S*)-3-(aminomethyl)-7-ethoxybenzo[*c*][1,2]oxaborol-1(3*H*)-ol (Figure 2) to carboxylic acid side chains of D447, D450, and the carbonyl group of M441. The 7-ethoxy substitution was important for packing with the Ade76 ribose, thus further stabilizing the boron-tRNA adduct. The research group concluded an extensive SAR study on the interaction with *Mtb* LeuRS using isothermal titration calorimetry. Halogen substitution (Cl/Br) in position 4 of benzoxaborole can enhance its affinity to the *Mtb* LeuRS editing domain [13]. In the following study, the authors concluded that the positions C-6 and C-7 are solvent-exposed and may be used for further structural modification to improve the selectivity. Large aromatic substitution in position 7 led to a decrease in antimycobacterial activity. For the modification of C-6, only aliphatic substitution was applied [14].

## 2. Results and Discussion

### 2.1. In Silico Studies

**Figure 3:**
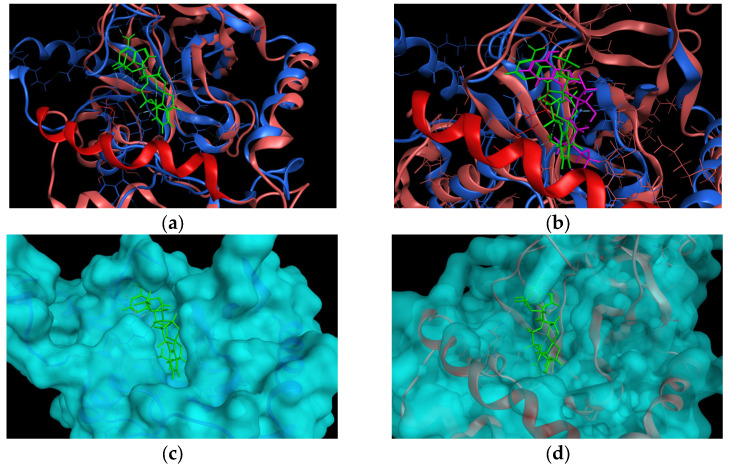
(**a**) Superimposition of 5AGR mtbLeuRS (blue color) with co-crystallized adduct (*S*)-3-(aminomethyl)-7-ethoxybenzo[*c*][1,2]oxaborol-1(3*H*)-ol-AMP as a ligand (light green color) and 2WFD hLeuRS (pink color) with highlighted additional alfa-helix (red color). (**b**) Superimposition of 5AGR mtbLeuRS (blue color) with ligand (green color) and 6LPF hLeuRS (pink color) with co-crystallized 2’ (L-norvalyl)amino-2’-deoxyadenosine and 5’-*O*-(L-leucylsulfamoyl)adenosine as a ligand (purple color) and highlighted additional alfa-helix (red color). (**c**) 5AGR mtbLeuRS with a calculated surface area of the enzyme (light blue color) and co-crystallized adduct (light green color). (**d**) 2WFD hLeuRS with a calculated surface area of the enzyme (light blue color) and co-crystallized adduct from 5AGR (light green color).

To explore the hypothesis on the difference between human cytosolic LeuRS and *Mtb* H37Rv LeuRS, we first superimposed crystallographic structures of the complex of *Mtb* H37Rv LeuRS (mtbLeuRS) editing domain (PDB ID: 5AGR deposited by Palencia et al. [13]) and human cytosolic LeuRS (hLeuRS) editing domain (PDB ID: 2WFD deposited by Seiradake et al. [17]). For the reason of the significant difference in the sequences of human and mycobacterial editing domains, the superimposition of the enzymes was performed according to the structural motifs and then the alignment of amino acid sequences was performed to match the superimposition, otherwise the superimposition was not successful. From the superimposition (Figure 3a), a significant difference in the binding sites of these two enzymes was observed. The binding pocket of the human cytosolic enzyme was closed over by an additional alfa-helix (R^457^EKLAEAKEKIYLKGFYE^474^), which was missing in the mycobacterial enzyme.

Due to the poor resolution of the crystallographic structure of 2WFD and poor sequence similarity of mtbLeuRS and hLeuRS, we decided to superimpose the 5AGR with another crystallographic structure of human cytosolic LeuRS co-crystallized with 2’-(L-norvalyl)amino-2’-deoxyadenosine and 5’-*O*-(L-leucylsulfamoyl)adenosine (PDB ID: 6LPF deposited by Liu et al. [30]) to check whether the position of the adenine core remains in the same position within the binding pocket of hLeuRS. The superimposition of 5AGR with 6LPF had to be performed according to the structural motifs as well. The superimposition of mtbLeuRS and the other human enzyme 6LPF (Figure 3b) revealed an analogic position of ligands in both enzymes and, again, an additional alfa-helix (R^457^EKLAEAKEKIYLKGFYE^474^) that closes over the pocket of human LeuRS was detected.

To better rendering the binding pocket, the surface area of the enzymes was calculated, which is depicted in Figure 3c for the mtbLeuRS 5AGR and Figure 3d for the hLeuRS 2WFD.

With the aim to investigate if the planned compounds (**1**–**19**) (Table 1) would fit in the mtbLeuRS and whether the large substitution would provide important additional interactions with mycobacterial enzyme, we ran docking studies. As our compounds were expected to act via the OBORT mechanism [12], we assumed that they should form a bivalent adduct with Ade76 and bound to the enzyme as the co-crystalized ligand does to 5AGR. Therefore, a library of bivalent adducts of the compounds with AMP was created with the stereochemistry corresponding to the co-crystalized ligand to mimic Ade76. To keep the interaction between the AMP moiety of the adduct and the enzyme unchanged, template docking was set with the AMP core and induced fit receptor. The score values for every unique molecule were in the range from −10.318074 to −8.267931 (Table S1).

**Figure 4:**
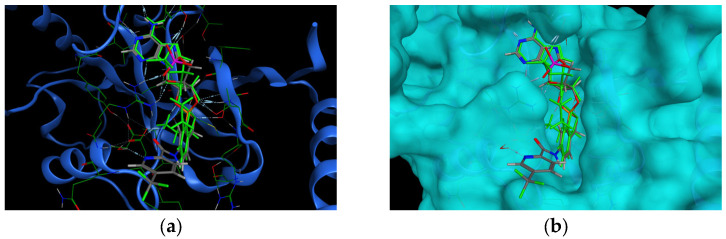
(**a**) The pose of one of the template-docked compounds, compound **11** (grey) into mtbLeuRS (PDB ID: 5AGR, the original co-crystallized ligand in green) interacting with D447. (**b**) The same pose with the calculated surface.

The retrieved poses matched the thesis, no steric clashes were observed that would indicate any problems with the binding mode of the mtbLeuRS (5AGR) with the compounds. Some pyrazine- and pyridine-substituted derivatives, that were bearing the nitrogen atom in the neighboring position in the (hetero)aryl moiety bound to the carboxamide group, exhibited specific H-bond interaction between the nitrogen atom of the heterocycle and D447 through a water molecule. The same amino acid residue formed one of the crucial interactions with the aminomethyl group in derivatives described by Palencia et al. [13] (Figure 4). In some cases, the aromatic substitution also exhibited an additional arene–H interaction with the hydrogen atom from T476.

**Figure 5:**
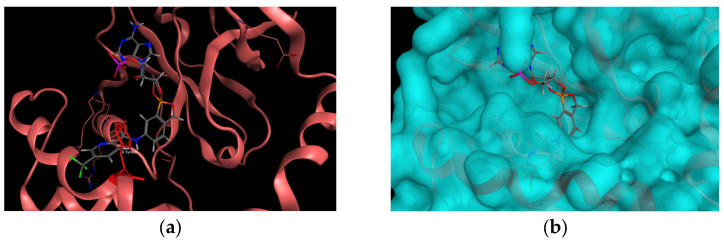
Superimposition of the hLeuRS (PDB ID: 2WFD) visualized with docking results of compound **11** (grey color). (**a**) 2WFD hLeuRS (pink color) visualized without calculated surface with highlighted Y468 (red color). (**b**) 2WFD hLeuRS visualized with the calculated surface.

To complete our thesis, the results from the template-docking into mtbLeuRS were compared with the hLeuRS (PDB ID: 2WFD) in a superimposition. It seems that the additional alfa-helix (R^457^EKLAEAKEKIY^468^LKGFYE^474^) in the human enzyme would cause serious steric clashes with the largely substituted compounds (Figure 5a), especially due to the presence of Y468, which is more obvious in the rendering with the calculated surface of 2WFD (Figure 5b).

Based on the computational studies, it can be concluded that the large aromatic substitution in position 6 of the benzoxaborole moiety should be beneficial for the selectivity of the compounds toward LeuRS of *Mtb* H37Rv and may provide some additional interactions, whereas the planned compounds should not be able to bind to the human LeuRS, as they should not be able to fit in the relatively small binding pocket.

### 2.2. Synthesis of the Compounds

The final compounds **1**–**19** (Table 1) were prepared by the acylation of 6-aminobenzo[*c*][1,2]oxaborol-1(3*H*)-ol hydrochloride with the corresponding activated (hetero)arylcarboxylic acid in an overall yield of 19–98%. The compounds have been characterized using melting points, ^1^H-NMR, ^13^C-NMR, ^11^B-NMR, IR, and MS spectra. Their purity was checked using elemental analysis or HPLC. In the ^13^C NMR spectra, the carbon attached to boron was usually not observed due to the quadrupolar relaxation of ^10^B and ^11^B nuclei. The ^13^C NMR spectrum of compound **8** was not obtained in sufficient quality due to the low solubility of the compound. The shift of the boron signal in the ^11^B-NMR spectra was found within the range of 30.36–35.07 ppm, which is in accordance with published data (32 ppm [4]).

In the IR spectra, a sharp absorption band was observed for valence vibration of the O-H bond at the frequencies 3460–3298 cm^−1^, which is comparable to the literature data 3400–3200 cm^−1^ [7] and 3450–3350 cm^−1^ [31] for benzoxaborole. For some compounds, a broad absorption band for valence vibration N-H bond in amide appeared in the range of 3376–3298 cm^−1^. Typical absorption bands were detected for all the compounds for the deformation vibration of carbonyl at the frequencies 1706–1631 cm^−1^ and for the deformation vibration of the N-H bond in amide at the frequencies 1541–1523 cm^−1^. Apart from the typical absorption band of the hydroxy group attached to benzoxaborole, there are two other typical bands, which are the absorbance of the C-O bond producing an intensive band of valency vibration at the frequencies 1001–956 cm^−1^ comparable to the literature value of 985–970 cm^−1^ [7] and 1005–970 cm^−1^ [31] and the absorbance of the B-O bond presenting at frequencies 1394–1361 cm^−1^ comparable to literature values of 1475–1375 cm^−1^ [7] and 1380–1340 cm^−1^ [31].

### 2.3. In Vitro Antimycobacterial Activity

#### 2.3.1. Antimycobacterial Activity against Collection Strains of Mycobacteria

All the synthesized compounds **1**–**19** were evaluated on their antimycobacterial activity using microplate Alamar Blue assay (MABA, [32]) against pathogenic *Mycobacterium tuberculosis* H37Rv CNCTC My 331/88 (ATCC 27294), avirulent strain *Mycobacterium tuberculosis* H37Ra ITM-M006710 (ATCC 9431), fast-growing *M. smegmatis* DSM 43,465 (ATCC 607), *M. aurum* DSM 43,999 (ATCC 23366), and non-tuberculous mycobacteria *M. avium* DSM 44,156 (ATCC 25291), *M. kansasii* DSM 44,162 (ATCC 12478). The most attractive compounds were also tested against multi-drug-resistant (MDR) clinical isolates of *Mtb*. The minimum inhibitory concentration (MIC) values were measured in µg/mL and then were recalculated into µM. The fast-growing *M. smegmatis* and *M. aurum* [33] (recently reclassified into genus *Mycolicibacterium* [34]) are commonly used as valid and safe non-pathogenic models for antimycobacterial research as an alternative to *Mtb* H37Rv [35,36,37].

Aryl or heteroaryl substitution bound in position 6 of benzoxaborole via the amidic linker-produced compounds active against the virulent strain *Mtb* H37Rv (Table 1) with few exceptions (compounds **6**, **7**, **13**, and **18**). Most of the compounds (**1**–**9**) were substituted with a pyrazine ring mimicking the clinically used anti-TB drug pyrazinamide and supported by our previous long-term experience with the synthesis of pyrazine-based derivatives [38,39,40]. However, other ring analogs with a pyridine ring (compounds **10**–**13**), a benzene ring (compound **14**), or even with a five-member aromatic ring (compounds **15** and **16**), and bicyclic aromatic systems (compounds **17**–**19**) have been prepared.

The most active compound among the pyrazine derivatives was the 5-chloro substituted compound **5** (MIC*_Mtb_*_H37Rv_ 21.59 µM). The high antimycobacterial activity of compound **5** can be explained by the higher lipophilicity due to the chlorine substitution and, thus, the better penetration through the mycolic cell wall of mycobacteria. The pyrazine ring should also have the ability to form H-bond interaction with D447 through a water molecule. In comparison to the 5-chloro benzene analog, the inhibitory activity was retained only against *M. aurum* (MIC around 15 µM). The change of the six-member aromatic ring for a five-member aromatic ring did not bring an increase in activity against *Mtb* H37Rv. Among the derivatives bearing aromatic nitrogen bicycles, the quinoxaline substitution produced compound **17** with MIC = 20.49 µM against *Mtb* H37Rv. The most active derivative against *Mtb* H37Rv in the whole series was 5-trifluoro-2-pyridyl derivative (**11**) with MIC = 9.72 µM, ruling the positional 2-trifluoro-5-pyridyl isomer (**12**, MIC = 155.26 µM) and the non-substituted 2-pyridyl derivative (**10**, MIC = 49.20 µM). Notably, compounds **1**, **2**, **5**, **10**, **11**, **14**, **15,** and **17** exhibited comparable or better activity against *Mtb* H37Rv than unsubstituted benzoxaborole moiety and compound **11** exhibited comparable inhibition to the activity of 3-aminomethylbenzoxaborole (both included in Table 1 and extracted from the publication by Palencia et al. [13]). Nevertheless, the activity of compound **11** did not overtake the MIC value of isoniazid (2.84 µM).

Regarding the comparison of inhibitory activity against *Mtb* H37Rv with the one against *Mtb* H37Ra, there was a satisfactory correlation with the exception of a few compounds (**3**, **8**, **11**, **14**, **17**, and **19**).

As for the SAR of the nitrogen-containing six-member ring, it can be concluded that a halogenated substitution in position 5 is favorable. 3-Amino substitution of the pyrazine ring led to a complete loss of inhibitory activity against *Mtb*. It can be generally stated that a lipophilic substitution is more convenient than any hydrophilic substitution (amino, hydroxy). However, no direct correlation has been found between the inhibitory activity against *Mtb* and the calculated lipophilicities (Table 1).

As for other tested mycobacterial strains, there is a good correlation between the activity of the compounds against *Mtb* H37Rv and *M. kansasii*. The most active derivatives against *M. kansasii* were the 5-trifluoro-2-pyridyl derivative (compound **11**, MIC 12.14 µM) and the quinoxalin-2-yl derivative (compound **17**, MIC 25.60 µM). An inverse effect was observed for the chlorinated pyrazine derivative. The more convenient location for chloro substitution was position 5 in the case of *Mtb* H37Rv inhibition, whereas position 6 (compound **4**) is more convenient for the inhibition of *M. kansasii* (MIC = 26.98 µM) and *M. avium* (MIC = 26.98 µM).

*M. avium* turned out to be the least susceptible strain to the tested compounds. The most active compound against *M. avium* proved to be the derivative with quinolin-2-yl substitution (compound **19**, MIC = 25.58 µM).

The rapidly growing mycobacteria *M. aurum* and *M. smegmatis* appeared to be the most susceptible strains. Compound **17** with the quinolin-2-yl substitution was the most active compound inhibiting *M. aurum* and *M. smegmatis* with identical MIC =12.82 µM. Generally, it can be stated that most of the compounds exhibited a broad spectrum of antimycobacterial activity also against non-tuberculous strains of mycobacteria in several cases even better than standard isoniazid. Compounds **3** and **17** proved comparable inhibition as rifampicin against *M. smegmatis*.

Compound **10** does not seem to show the best growth inhibition, but it should be pointed out from the series, as it inhibited the growth of all mycobacterial strains (MIC below 62 µM).

Some SAR phenomena can be explained by docking studies. Pyrazine and pyridine derivatives were able to bind to a molecule of water and the D447 residue to form an H-bond interaction between the heterocyclic nitrogen and an editing domain of mycobacterial LeuRS. This interaction could not be observed for the benzene derivatives, which is in accordance with the lower activity of these derivatives. Focusing on the two position isomers **11** and **12**, the higher activity of 5-trifluoro-2-pyridyl (**11**) can be explained by the sufficient proximity of nitrogen for hydrogen bond formation, whereas the nitrogen in 2-trifluoro-5-pyridyl derivative (**12**) is too far for the formation of the additional hydrogen bond. The same correlation has been found for the pair of compounds **9** (4-hydroxypyrazin-2-yl derivative) and **13** (2-hydroxy-5-pyridyl derivative). Only compound **9** was able to form the hydrogen bond, which was confirmed in the docking studies, and it also proved higher inhibitory activity against *Mtb* H37Rv (MIC = 184.48 µM) compared to compound **13** (MIC > 370.30 µM).

#### 2.3.2. Antimycobacterial Activity against Multi-Drug-Resistant Clinical Isolates of *M. tuberculosis*

The promising candidates, namely compounds **4**, **5**, **10**, and **17**, were also evaluated against multidrug-resistant (MDR) clinical isolates of *Mtb*. The used MDR isolates were resistant to streptomycin and the majority of first-line anti-TB drugs such as isoniazid, rifampicin, and pyrazinamide. In Table 2, the consistent results of the prepared compounds against *Mtb* H37Rv and MDR strains are demonstrated. There is just a slight decrease in the activity against MDR strains (one dilution difference), which indicates a unique mechanism of action that is not related to any of the first-line anti-TB drugs. The complete resistance profile of the MDR strains can also be found in Section 3.3.1 (antimycobacterial screening against *Mtb* H37Rv and MDR strains of *Mtb*).

### 2.4. In Vitro Antibacterial and Antifungal Evaluation

Benzoxaborole moiety itself exerts a broad spectrum of inhibitory activity against several strains of fungi [41]. Its simple derivatives may affect the viability of several strains of bacteria. So, the problem in designing new benzoxaborole derivatives is not just in obtaining compounds with high activity but also obtaining compounds with enough selectivity. Therefore, the series of title compounds were evaluated on activities against a set of sixteen microorganisms, eight bacterial strains, and eight fungal strains. A microdilution method according to EUCAST [42,43,44] was used. The MIC values were expressed in µM. The derivatives **7**, **11**, **14**, **18**, and **19** were tested up to the concentration of 125 µM, while the rest were tested up to the concentration of 500 µM. For the methodology and the complete list of tested strains, please see the Supplementary Materials.

None of the tested compounds proved any inhibitory activity at the tested concentrations against the included strains of fungi and yeasts (Table S4). This may be explained by the difference in the structure between prokaryotic and eukaryotic LeuRS and by the steric clash with the extra alfa helix discussed herein in the computational studies. Most of the compounds did not exert any inhibitory activity against Gram-negative strains of bacteria (Table S2) besides compound **16**, which proved very weak inhibition (MIC = 500 µM) after 24 h incubation and no inhibition after 48 h incubation against *Escherichia coli* and *Klebsiella pneumoniae*. In the case of a few compounds, very weak inhibitory activity was determined against Gram-positive bacteria with MIC values in the concentration range of 125–250 µM. This led us to the conclusion that the title compounds selectively affect only strains of mycobacteria and mycolicibacteria.

### 2.5. Cytotoxicity Screening

The cytotoxicity of the most promising compounds was evaluated using standard human liver cancer cell line HepG2 in a commercial CellTiter 96 assay. The determined IC_50_ values allowing a quantitative comparison of the toxicity among the tested compounds are presented in Table 1. Several established anti-tuberculosis drugs are known to manifest a risk of hepatotoxic behavior [45] and the cytotoxic effect on the hepatic cell line is thus a relevant surrogate.

The majority of the tested compounds were not toxic and no significant decrease in cell viability was observed in the highest tested concentrations (IC_50_ > 1000 µM). For seven compounds with solubility issues in the incubation medium, the IC_50_ values were determined in the highest concentrations possible. The derivatives **2** and **3** were determined up to the concentration of 500 µM, compounds **12** and **17** up to the concentration of 250 µM, and compounds **6**, **7**, and **11** up to the concentration of 100 µM. All appeared to be non-toxic up to their highest tested concentrations.

The selectivity of the most active compounds Is expressed as a selectivity index (SI = IC_50_ (µM)/MIC*_Mtb_*_H37Rv_ (µM)) (Table 3). For the majority of compounds, the SI value is above 10, which can be considered a reasonable starting point for further optimization. Only compounds **5** and **15** proved to have SI values below 5.

## 3. Materials and Methods

### 3.1. In Silico Study

Docking studies were performed using Dock utility in MOE 2022.02 (Chemical Computing Group, Montreal, QC, Canada), using the AMBER10:EHT force field. The editing domain of mtbLeuRS (PDB ID: 5AGR), the editing domain of hLeuRS (PDB ID: 2WFD), and human cytosolic LeuRS (hLeuRS2, PDB ID: 6LPF) were downloaded from the RCSB PDB database. 5AGR was firstly adjusted by removing artifacts from crystallography, namely Met-Leu and EDO (1,2-ethanediol) in the sequence editor. Then, solvent molecules were erased, besides the ones that formed H-bond interaction with amino acid residues. 2WFD was adjusted by the deletion of the additional B chain in the sequence editor. 6LPF was adjusted in the sequence editor by removing the A chain of the dimer, several ligands (LSS, GOL), and solvent molecules. The following enzymes were superposed: 5AGR with 2WFD and 5AGR with 6LPF. The position of 5AGR was firstly fixed and then the superposition of enzymes according to their structural motifs and subsequently, the alignment of the sequences was performed to match the superposition, otherwise, the superposition was not successful. All the proteins or complexes were prepared for subsequent docking using the function QuickPrep (with default settings). The bivalent adducts of synthesized compounds and AMP were drawn in ChemDraw 20.0 (PerkinElmer Informatics, Waltham, MA, USA). After import into MOE, the structures were prepared (choice of protonation state at pH 7, calculation of partial charges, and energy minimization until RMS = 0.00001 kcal.mol^−1^Å^−1^). The adducts of synthesized compounds were docked in the 5AGR using the template docking utility with the AMP moiety defined as the template. The superposed hLeuRS1 and hLeuRS2 were inactivated during docking. The thirty best poses from placement were refined to the final two poses for every entry (minimized inside the induced fit receptor) and scored using the GBVI/WSA dG scoring function. The resulting poses were sorted according to the ascending score value and the poses were analyzed visually. The score values for every unique molecule are involved in Table S1.

### 3.2. Synthesis of the Compounds

#### 3.2.1. General Information

All the reagents and solvents (unless stated otherwise) were purchased from Sigma-Aldrich (St. Louis, MO, USA) or Fluorochem (Hadfield, Derbyshire, UK) and used without further purification. The reaction progress and purity of the products were monitored using thin-layer chromatography (TLC) using Silica gel 60 F_254_ sheets (Merck, Darmstadt, Germany). The flash chromatography of some final compounds was performed using PuriFlash XS 420+ (Interchim, Montluçon, France) with original columns (spherical silica, particle size 30 µm) with detection using UV-VIS detection at 254 nm and 280 nm. The elution ran in isocratic mode using a mobile phase consisting of hexane (Hex), ethyl acetate (EtOAc), and methanol (MeOH) at a volume ratio of 40:55:5. The NMR spectra were recorded using a Varian VNMR S500 (Varian, Palo Alto, CA, USA) at 500 MHz for ^1^H, 126 MHz for ^13^C, and 160 MHz for ^11^B, and the ^1^H and ^13^C spectra of compounds **3, 9**–**14, 16, 18,** and **19** using Jeol JNM-ECZ600R (Jeol Ltd., Tokyo, Japan) at 600 MHz for ^1^H and 151 MHz for ^13^C. The spectra were measured in DMSO-*d*6 at ambient temperature. The chemical shifts reported as δ values in ppm indirectly referred to tetramethylsilane (TMS) via the solvent signal (2.49 for ^1^H and 39.7 for ^13^C in DMSO-*d*6). The IR spectra were recorded using an NICOLET 6700 FT-IR spectrophotometer (Thermo Scientific, Waltham, MA, USA) using the ATR-Ge method. The elemental analyses were measured using a Vario Micro Cube Elemental Analyzer (Elementar Analysensysteme, Hanau, Germany) and the values are provided in percentage. The yields are provided in percentage and refer to the amount of pure product after all the purification steps. The melting points were determined in open capillary on a Stuart SMP30 melting point apparatus (Bibby Scientific Limited, Staffordshire, UK) and are uncorrected. The purities of the studied compounds were determined using Agilent Technologies 1200 SL liquid chromatograph (Agilent Technologies, Santa Clara, CA, USA) consisting of a vacuum microdegasser, 1200 SL binary pump, 1200 SL plus autosampler, TCC Infinity 1290 column thermostat, and 1200 SL diode-array detector. The chromatographic system was controlled using an Agilent ChemStation (Agilent Technologies, Santa Clara, CA, USA), version B.04.02 extended by a spectral module. The area percentage method at the wavelength 250 nm was applied where it was proven to have >95% purity. The Log*P* values were calculated using ChemDraw 20.0 part of ChemOffice 2020 package (PerkinElmer Informatics, Waltham, MA, USA). The mass spectra in both positive and negative mode (APCI-MS) were measured using the Expression^®^ Compact Mass Spectrometer (Advion, Ithaca, NY, USA) with a single-quad detector. The samples were applied as solids by the ASAP probe method (Advion, Ithaca, NY, USA).

#### 3.2.2. General Synthetic Procedure

In the first step, the corresponding (hetero)arylcarboxylic acid (2 mmol) was activated with 1,1’-carbonyldiimidazole (CDI; 2.2 mmol) in DMSO (5 mL) for 1 h. After initial heating, it proceeded at room temperature. Then, 6-aminobenzo[*c*][1,2]oxaborol-1(3*H*)-ol hydrochloride (2 mmol) dissolved in DMSO (10 mL) was added and the reaction mixture was stirred at room temperature overnight. The working-up was accomplished by the addition of 1M HCl (10 mL). Within one hour of stirring, precipitation appeared. The precipitate was filtrated off and washed with water. All the final compounds were dried over anhydrous phosphorus pentoxide under reduced pressure at room temperature before characterization and purity check. Compounds **10** and **15** were not completely clean so we further purified them using Flash chromatography using isocratic elution with the mobile phase consisting of Hex:EtOAc:MeOH = 4:5.5:0.5. The compounds demonstrated R_f_ values of 0.85 and 0.9, respectively, in this mobile phase.

#### 3.2.3. Characterization and Purity of the Compounds **1**–**19**

In the ^1^H-NMR spectra, the following abbreviations have been used: PzH—pyrazine hydrogen, PyH—pyridine hydrogen, QxH—quinoxaline hydrogen, TzH—thiazole hydrogen, and LmH—hydrogen involved in lactam-lactim tautomerism. In the ^13^C-NMR spectra, the following abbreviations have been used: PyC—pyridine carbon. The ^1^H-NMR, ^13^C-NMR, and ^11^B-NMR spectra of the title compounds with solvent signals omitted are involved.

***N*-(1-hydroxy-1,3-dihydrobenzo[*c*][1,2]oxaborol-6-yl)pyrazine-2-carboxamide (1).** Light yellow solid. Yield: 79%. mp 261.3–262.3 °C. ^1^H NMR (500 MHz, dmso) δ 10.75 (s, 1H, CONH), 9.30 (d, *J* = 1.5 Hz, 1H, PzH), 9.26 (s, 1H, OH), 8.92 (d, *J* = 2.5 Hz, 1H, PzH), 8.80 (dd, *J* = 2.5, 1.5 Hz, 1H, PzH), 8.28 (d, *J* = 2.0 Hz, 1H, ArH), 7.85 (dd, *J* = 8.3, 2.0 Hz, 1H, ArH), 7.42–7.37 (m, 1H, ArH), 4.97 (s, 2H, CH_2_). ^13^C NMR (126 MHz, dmso) δ 161.89, 150.01, 147.86, 145.38, 144.25, 143.45, 137.14, 131.15, 124.08, 122.72, 121.77, 69.96. ^11^B NMR (160 MHz, dmso) δ 31.62. IR (ATR-Ge, cm^−1^): 3317 (ν O-H), 1672 (δ, C=O), 1535 (δ CONH), 1369 (ν B-O), 990 (ν C-O ether). Analysis calculated for C_12_H_10_BN_3_O_3_ (M_r_ 255.04): C, 56.51; H, 3.95; N, 16.48. Found: C, 56.30; H, 3.65; N, 16.48. MS: [M + H]^+^ = 255.9 (exact mass 255.08).

***N*-(1-hydroxy-1,3-dihydrobenzo[*c*][1,2]oxaborol-6-yl)-5-methylpyrazine-2-carboxamide (2).** Light yellow solid. Yield: 72%. mp 215.0–216.1 °C. ^1^H NMR (500 MHz, dmso) δ 10.67 (s, 1H, CONH), 9.24 (s, 1H, OH), 9.15 (d, *J* = 1.5 Hz, 1H, PzH), 8.68 (d, *J* = 1.5 Hz, 1H, PzH), 8.28 (d, *J* = 2.0 Hz, 1H, ArH), 7.84 (dd, *J* = 8.2, 2.0 Hz, 1H, ArH), 7.41–7.35 (m, 1H, ArH), 4.96 (s, 2H, CH_2_), 2.62 (s, 3H, CH_3_). ^13^C NMR (126 MHz, dmso) δ 162.26, 157.58, 150.12, 143.46, 143.18, 142.86, 137.47, 131.38, 124.29, 122.91, 121.97, 70.20, 21.88. ^11^B NMR (160 MHz, dmso) δ 33.75. IR (ATR-Ge, cm^−1^): 3319 (ν O-H), 1673 (δ, C=O), 1537 (δ CONH), 1367 (ν B-O), 993 (ν C-O ether). Analysis calculated for C_13_H_12_BN_3_O_3_ (M_r_ 269.07): C, 58.03; H, 4.50; N, 15.62. Found: C, 57.80; H, 4.23; N, 15.60. MS: [M + H]^+^ = 269.9 (exact mass 269.10).

***N*-(1-hydroxy-1,3-dihydrobenzo[*c*][1,2]oxaborol-6-yl)-3-methylpyrazine-2-carboxamide (3).** Light white solid. Yield: 72%. mp 262.9–264.0 °C. ^1^H NMR (600 MHz, dmso) δ 10.66 (s, 1H, CONH), 9.24 (brs, 1H, OH), 8.72 (d, *J* = 2.5 Hz, 1H, PzH), 8.60 (dd, *J* = 2.5, 0.8 Hz, 1H, PzH), 8.26 (d, *J* = 2.0 Hz, 1H, ArH), 7.76 (dd, *J* = 8.2, 2.0 Hz, 1H, ArH), 7.39 (dd, *J* = 8.2, 0.9 Hz, 1H, ArH), 4.96 (s, 2H, CH_2_), 2.76 (d, *J* = 0.6 Hz, 3H, CH_3_). ^13^C NMR (151 MHz, dmso) δ 164.39, 153.49, 150.10, 146.18, 146.12, 141.33, 137.87, 131.54, 123.83, 122.47, 122.15, 70.27, 22.93. ^11^B NMR (160 MHz, dmso) δ 32.51. IR (ATR-Ge, cm^−1^): 3344 (ν O-H), 3068 (ν C-H arom.), 1683 (δ, C=O), 1525 (δ CONH), 1373 (ν B-O), 978 (ν C-O ether). Analysis calculated for C_13_H_12_BN_3_O_3_ (M_r_ 269.07): C, 58.03; H, 4.5; N, 15.62. Found: C, 57.58; H, 4.22; N, 15.18. MS: [M + H]^+^ = 270.0 (exact mass 269.10).

**6-chloro-*N*-(1-hydroxy-1,3-dihydrobenzo[*c*][1,2]oxaborol-6-yl)pyrazine-2-carboxamide (4).** White solid. Yield: 66%. mp 250.9–252.4 °C. ^1^H NMR (500 MHz, dmso) δ 10.68 (s, 1H, CONH), 9.26 (s, 1H, OH), 9.23 (s, 1H, PzH), 9.05 (s, 1H, PzH), 8.24 (d, *J* = 2.0 Hz, 1H, ArH), 7.82 (dd, *J* = 8.2, 2.0 Hz, 1H, ArH), 7.40 (d, *J* = 8.2 Hz, 1H, ArH), 4.97 (s, 2H, CH_2_). ^13^C NMR (126 MHz, dmso) δ 160.85, 150.23, 147.56, 147.10, 145.46, 142.52, 136.91, 131.14, 124.29, 123.04, 121.77, 69.96.^11^B NMR (160 MHz, dmso) δ 32.52. IR (ATR-Ge, cm^−1^): 3368 (ν O-H), 3057 (ν C-H arom.), 1681 (δ, C=O), 1523 (δ CONH), 1367 (ν B-O), 992 (ν C-O ether). Analysis calculated for C_12_H_9_BClN_3_O_3_ (M_r_ 289.48): C, 49.79; H, 3.13; N, 14.52. Found: C, 49.32; H, 2.87; N, 14.38. MS: [M + H]^+^ = 289.9 (exact mass 289.04).

**5-chloro-*N*-(1-hydroxy-1,3-dihydrobenzo[*c*][1,2]oxaborol-6-yl)pyrazine-2-carboxamide (5).** White solid. Yield: 41%. mp 233.8–235.3 °C. ^1^H NMR (500 MHz, dmso) δ 10.77 (s, 1H, CONH), 9.25 (brs, 1H, OH), 9.11 (d, *J* = 1.4 Hz, 1H, PzH), 8.92 (d, *J* = 1.4 Hz, 1H, PzH), 8.26 (d, *J* = 2.1 Hz, 1H, ArH), 7.83 (dd, *J* = 8.2, 2.1 Hz, 1H, ArH), 7.39 (d, *J* = 8.2 Hz, 1H, ArH), 4.96 (s, 2H, CH_2_). ^13^C NMR (126 MHz, dmso) δ 161.34, 151.32, 150.37, 144.47, 144.41, 143.39, 137.29, 124.41, 123.08, 122.02, 70.21. ^11^B NMR (160 MHz, dmso) δ 31.95. IR (ATR-Ge, cm^−1^): 3355 (ν O-H), 1693 (δ, C=O), 1542 (δ CONH), 1369 (ν B-O), 978 (ν C-O ether). Analysis calculated for C_12_H_9_BClN_3_O_3_ (M_r_ 289.48): C, 49.79; H, 3.13; N, 14.52. Found: C, 50.16; H, 2.96; N, 14.41. MS: [M + H]^+^ = 289.9 (exact mass 289.04).

**3-amino-*N*-(1-hydroxy-1,3-dihydrobenzo[*c*][1,2]oxaborol-6-yl)pyrazine-2-carboxamide (6).** Yellow solid. Yield: 98%. mp 246.6–249.4 °C. ^1^H NMR (500 MHz, dmso) δ 10.52 (s, 1H, CONH), 9.23 (s, 1H, OH), 8.27 (d, *J* = 2.3 Hz, 1H, PzH), 8.20 (d, *J* = 2.1 Hz, 1H, ArH), 7.92 (d, *J* = 2.3 Hz, 1H, PzH), 7.77 (dd, *J* = 8.2, 2.1 Hz, 1H, ArH), 7.59 (brs, 2H, NH_2_), 7.37 (d, *J* = 8.2 Hz, 1H, ArH), 4.96 (s, 2H, CH_2_). ^13^C NMR (126 MHz, dmso) δ 164.77, 155.67, 149.71, 147.51, 137.13, 131.22, 125.67, 124.09, 122.58, 121.73, 69.97. ^11^B NMR (160 MHz, dmso) δ 32.21. IR (ATR-Ge, cm^−1^): 3409 (ν O-H), 3337 and 3299 (NH_2_), 1672 (δ, C=O), 1542 (δ CONH), 1367 (ν B-O), 986 (ν C-O ether). Analysis calculated for C_12_H_11_BN_4_O_3_ (M_r_ 270.06): C, 53.37; H, 4.11; N, 20.75. Found: C, 52.93; H, 3.73; N, 20.39. MS: [M + H]^+^ = 270.9 (exact mass 270.09).

**3-amino-6-chloro-*N*-(1-hydroxy-1,3-dihydrobenzo[*c*][1,2]oxaborol-6-yl)pyrazine-2-carboxamide (7).** Yellow solid. Yield: 53%. mp 242.9–244.4 °C. ^1^H NMR (500 MHz, dmso) δ 10.33 (s, 1H, CONH), 9.21 (s, 1H, OH), 8.37 (s, 1H, PzH), 8.15 (d, *J* = 2.1 Hz, 1H, ArH), 7.76 (dd, *J* = 8.2, 2.1 Hz, 1H, ArH), 7.72 (brs, 2H, NH_2_), 7.38 (d, *J* = 8.2 Hz, 1H, ArH), 4.96 (s, 2H, CH_2_). ^13^C NMR (126 MHz, dmso) δ 164.02, 154.72, 150.19, 147.07, 137.12, 132.19, 124.64, 124.50, 123.31, 121.94, 70.22. ^11^B NMR (160 MHz, dmso) δ 32.03. IR (ATR-Ge, cm^−1^): 3440 (ν O-H), 3348 and 3325 (NH_2_), 1670 (δ, C=O), 1535 (δ CONH), 1362 (ν B-O), 977 (ν C-O ether). Analysis calculated for C_12_H_10_BClN_4_O_3_ (M_r_ 304.5): C, 47.33; H, 3.31; N, 18.4. Found: C, 47.42; H, 3.05; N, 18.39. MS: [M + H]^+^ = 305.0 (exact mass 304.05).

***N*-(1-hydroxy-1,3-dihydrobenzo[*c*][1,2]oxaborol-6-yl)-3-oxo-3,4-dihydropyrazine-2-carboxamide (8).** Light green solid. Yield: 53%. mp 326.4–328.3 °C (decomp.). ^1^H NMR (500 MHz, dmso) δ 13.23 (brs, 1H, LmH), 11.46 (s, 1H, CONH), 9.25 (brs, 1H, OH), 8.12 (d, *J* = 2.0 Hz, 1H, PzH), 7.82 (s, 1H, ArH), 7.75 (dd, *J* = 8.2, 2.1 Hz, 1H, ArH), 7.73–7.69 (m, 1H, PzH), 7.39 (d, *J* = 8.2 Hz, 1H, ArH), 4.96 (s, 2H, CH_2_). ^11^B NMR (160 MHz, dmso) δ 33.88. IR (ATR-Ge, cm^−1^): 3460 (ν O-H), 3040 (ν C-H arom.), 1694 (δ, C=O), 1652 (δ CONH), 1368 (ν B-O), 956 (ν C-O ether). Analysis calculated for C_12_H_10_BN_3_O_4_ (M_r_ 271.04): C, 53.18; H, 3.72; N, 15.50. Found: C, 52.79; H, 3.39; N, 15.36. MS: [M + H]^+^ = 271.8 (exact mass 271.08).

**5-hydroxy-*N*-(1-hydroxy-1,3-dihydrobenzo[*c*][1,2]oxaborol-6-yl)pyrazine-2-carboxamide (9).** Light brown solid. Yield: 93%. mp 338.3–340.1 °C. ^1^H NMR (600 MHz, dmso) δ 12.84 (brs, 1H, OH), 10.15 (s, 1H, CONH), 9.18 (brs, 1H, OH), 8.20 (d, *J* = 3.7 Hz, 1H, ArH), 8.09 (m, 1H, PzH), 8.05–8.01 (m, 1H, PzH), 7.80–7.74 (m, 1H, ArH), 7.37–7.32 (m, 1H, ArH), 4.96–4.93 (m, 2H, CH_2_). ^13^C NMR (151 MHz, dmso) δ 161.68, 156.80, 149.82, 146.83, 137.77, 131.26, 127.04, 124.21, 122.76, 121.96, 70.26. ^11^B NMR (160 MHz, dmso) δ 32.73. IR (ATR-Ge, cm^−1^): 3357 (ν O-H), 2796 (NH, Lactam), 1677 (δ, C=O), 1659 (CO, CONH), 1529 (δ CONH), 1367 (ν B-O), 968 (ν C-O ether). Calculated for C_12_H_10_BN_3_O_4_ (M_r_ 271.04): C, 53.18; H, 3.72; N, 15.5. Found: C, 52.74; H, 3.45; N, 15.10. MS: [M + H]^+^ = 271.8 (exact mass 271.08).

***N*-(1-hydroxy-1,3-dihydrobenzo[*c*][1,2]oxaborol-6-yl)picolinamide (10).** Yellow solid. Yield: 80%. mp 183.7–186.1 °C. ^1^H NMR (600 MHz, dmso) δ 10.64 (s, 1H, CONH), 9.23 (s, 1H, OH), 8.73 (m, 1H, PyH), 8.30 (d, *J* = 2.0 Hz, 1H, ArH), 8.16 (m, 1H, PyH), 8.06 (m, 1H, PyH), 7.85 (dd, *J* = 8.2, 2.0 Hz, 1H, ArH), 7.67 (m, 1H, PyH), 7.42–7.35 (m, 1H, ArH), 4.97 (s, 2H, CH_2_). ^13^C NMR (151 MHz, dmso) δ 163.00, 150.54, 150.03, 148.98, 138.66, 137.68, 131.50, 127.41, 124.16, 122.90, 122.68, 122.08, 70.29. ^11^B NMR (160 MHz, dmso) δ 31.61. IR (ATR-Ge, cm^−1^): 3311 (ν O-H), 1677 (δ, C=O), 1540 (δ CONH), 1366 (ν B-O), 980 (ν C-O ether). Calculated for C_13_H_11_BN_2_O_3_ (M_r_ 254.05): C, 61.46; H, 4.36; N, 11.03. Found: C, 61.19; H, 3.99; N, 10.99. MS: [M + H]^+^ = 255.0 (exact mass 254.09); R_f_ (Hex:EtOAc:MeOH = 4:5.5:0.5) = 0.85.

***N*-(1-hydroxy-1,3-dihydrobenzo[*c*][1,2]oxaborol-6-yl)-5-(trifluoromethyl)picolinamide (11).** Light yellow solid. Yield: 82%. mp 188.1–188.9 °C. ^1^H NMR (600 MHz, dmso) δ 10.79 (s, 1H, CONH), 9.24 (s, 1H, OH), 9.10 (m, 1H, PyH), 8.50–8.45 (m, 1H, PyH), 8.34 (m, 1H, PyH), 8.30 (d, *J* = 2.0 Hz, 1H, ArH), 7.86 (dd, *J* = 8.2, 2.0 Hz, 1H, ArH), 7.40 (dd, *J* = 8.2, 0.8 Hz, 1H, ArH), 4.97 (s, 2H, CH_2_). ^13^C NMR (151 MHz, dmso) δ 161.95, 154.13, 150.38, 145.83, 137.42, 136.31, 131.52, 127.92 (q, *J* = 32.5 Hz, 1C, PyC), 124.37, 123.92 (q, *J* = 273.8 Hz, 1C, CF_3_), 123.30, 122.98, 122.11, 70.29. ^11^B NMR (160 MHz, dmso) δ 30.36. IR (ATR-Ge, cm^−1^): 3343 (ν O-H), 1682 (δ, C=O), 1531 (δ CONH), 1366 (ν B-O), 978 (ν C-O ether). HPLC purity 95.1 %. MS: [M + H]^+^ = 323.1 (exact mass 322.07).

***N*-(1-hydroxy-1,3-dihydrobenzo[*c*][1,2]oxaborol-6-yl)-6-(trifluoromethyl)nicotinamide (12).** Light yellow solid. Yield: 87%. mp 227.3–229.4 °C. ^1^H NMR (600 MHz, dmso) δ 10.67 (s, 1H, CONH), 9.26 (s, 1H, OH), 9.25 (d, *J* = 2.1 Hz, 1H, PyH), 8.57 (dd, *J* = 8.1, 2.1 Hz, 1H, PyH), 8.17 (d, *J* = 2.0 Hz, 1H, ArH), 8.09 (dd, *J* = 8.1, 0.9 Hz, 1H, PyH), 7.78 (dd, *J* = 8.2, 2.0 Hz, 1H, ArH), 7.43–7.39 (m, 1H, ArH), 4.98 (s, 2H, CH_2_). ^13^C NMR (151 MHz, dmso) δ 163.38, 150.35, 149.85, 148.67 (q, *J* = 34.1 Hz, 1C, PyC), 138.40, 137.88, 134.38, 131.57, 124.13, 122.98, 122.16, 121.95 (q, *J* = 273.9 Hz, 1C, CF_3_), 121.14, 70.29. ^11^B NMR (160 MHz, dmso) δ 32.24. IR (ATR-Ge, cm^−1^): 3298 (ν CONH), 1649 (δ, C=O), 1524 (δ CONH), 1372 (ν B-O), 985 (ν C-O ether). HPLC purity 95.0 %. MS: [M + H]^+^ = 323.1 (exact mass 322.07).

**6-hydroxy-*N*-(1-hydroxy-1,3-dihydrobenzo[*c*][1,2]oxaborol-6-yl)nicotinamide (13).** Beige solid. Yield: 28%. mp 304.8–306.9 °C. ^1^H NMR (600 MHz, dmso) δ 12.07 (brs, 1H, OH), 10.00 (s, 1H, CONH), 9.20 (s, 1H, OH), 8.19 (d, *J* = 2.7 Hz, 1H, PyH), 8.07 (d, *J* = 2.0 Hz, 1H, ArH), 7.97 (dd, *J* = 9.6, 2.7 Hz, 1H, PyH), 7.69 (dd, *J* = 8.2, 2.0 Hz, 1H, ArH), 7.35 (d, *J* = 8.2 Hz, 1H, ArH), 6.40 (d, *J* = 9.6 Hz, 1H, PyH), 4.95 (s, 2H, CH_2_). ^13^C NMR (151 MHz, dmso) δ 163.10, 162.90, 149.60, 139.90, 138.52, 138.34, 131.37, 124.09, 122.85, 121.95, 119.69, 113.17, 70.25. ^11^B NMR (160 MHz, dmso) δ 33.55. IR (ATR-Ge, cm^−1^): 3457 (ν O-H), 3131 (ν C-H arom.), 2918 (ν CH_2_), 2868 (NH, Lactam), 1662 (δ, C=O), 1556 (δ CONH), 1394 (ν B-O), 1001 (ν C-O ether). Calculated for C_13_H_11_BN_2_O_4_ (M_r_ 270.05): C, 57.82; H, 4.11; N, 10.37. Found: C, 57.39; H, 4.35; N, 9.95. MS: [M + H]^+^ = 270.9 (exact mass 270.08).

**4-chloro-*N*-(1-hydroxy-1,3-dihydrobenzo[*c*][1,2]oxaborol-6-yl)benzamide (14).** White solid. Yield: 92%. mp 225.5–228.1 °C. ^1^H NMR (600 MHz, dmso) δ 10.34 (s, 1H, CONH), 9.22 (s, 1H, OH), 8.16 (d, *J* = 2.0 Hz, 1H, ArH), 8.02–7.97 (m, 2H, ArH), 7.75 (dd, *J* = 8.1, 2.0 Hz, 1H, ArH), 7.62–7.57 (m, 2H, ArH), 7.38 (d, *J* = 8.1 Hz, 1H, ArH), 4.96 (s, 2H, CH_2_). ^13^C NMR (151 MHz, dmso) δ 164.95, 149.93, 138.27, 136.88, 134.23, 131.43, 130.17, 128.99, 124.26, 123.04, 122.00, 70.28. ^11^B NMR (160 MHz, dmso) δ 35.07. IR (ATR-Ge, cm^−1^): 3298 (ν O-H), 1638 (δ, C=O), 1536 (δ CONH), 1366 (ν B-O), 982 (ν C-O ether). Calculated for C_14_H_11_BClNO_3_ (M_r_ 287.51): C, 58.49; H, 3.86; N, 4.87. Found: C, 58.43; H, 3.64; N, 4.85. MS: [M + H]^+^ = 287.9 (exact mass 287.05).

**5-chloro-*N*-(1-hydroxy-1,3-dihydrobenzo[*c*][1,2]oxaborol-6-yl)thiophene-2-carboxamide (15).** Light yellow solid. Yield: 21%. mp 234.9–237.1 °C. ^1^H NMR (500 MHz, dmso) δ 10.35 (s, 1H, CONH), 9.24 (s, 1H, OH), 8.07 (d, *J* = 2.0 Hz, 1H, ArH), 7.92 (d, *J* = 4.1 Hz, 1H, TfH), 7.71 (dd, *J* = 8.2, 2.0 Hz, 1H, ArH), 7.39 (dd, *J* = 8.2, 0.8 Hz, 1H, ArH), 7.26 (d, *J* = 4.1 Hz, 1H, TfH), 4.96 (s, 2H, CH_2_). ^13^C NMR (126 MHz, dmso) δ 159.27, 150.03, 139.70, 137.54, 134.27, 129.50, 128.74, 124.15, 123.00, 122.06, 70.20. ^11^B NMR (160 MHz, dmso) δ 30.49. IR (ATR-Ge, cm^−1^): 3309 (ν O-H), 1631 (δ, C=O), 1541 (δ CONH), 1365 (ν B-O), 982 (ν C-O ether). Analysis calculated for C_12_H_9_BClNO_3_S (M_r_ 293.53): C, 49.10; H, 3.09; N, 4.77; S, 10.92. Found: C, 49.02; H, 2.95; N, 4.77; S, 11.26. MS: [M + H]^+^ = 293.9 (exact mass 293.01); R_f_ (Hex:EtOAc:MeOH = 4:5.5:0.5) = 0.9.

***N*-(1-hydroxy-1,3-dihydrobenzo[*c*][1,2]oxaborol-6-yl)thiazole-4-carboxamide (16).** Light yellow solid. Yield: 86%. mp 225.7–227.9 °C. ^1^H NMR (600 MHz, dmso) δ 10.33 (s, 1H, CONH), 9.26 (d, *J* = 2.1 Hz, 1H, ArH), 9.22 (brs, 1H, OH), 8.49 (d, *J* = 1.8 Hz, 1H, TzH), 8.22 (d, *J* = 1.8 Hz, 1H, TzH), 7.80 (dd, *J* = 8.2, 2.1 Hz, 1H, ArH), 7.37 (d, *J* = 8.2 Hz, 1H, ArH), 4.96 (s, 2H, CH_2_). ^13^C NMR (151 MHz, dmso) δ 159.67, 155.58, 151.33, 149.99, 137.78, 131.42, 125.93, 124.35, 122.96, 122.00, 70.28. ^11^B NMR (160 MHz, dmso) δ 33.03. IR (ATR-Ge, cm^−1^): IR 3373 (ν O-H), 3019 (ν C-H arom.), 2918 and 2868 (ν CH_2_), 1664 (δ, C=O), 1538 (δ CONH), 1361 (ν B-O), 977 (ν C-O ether). Analysis calculated for C_11_H_9_BN_2_O_3_S (M_r_ 260.07): C, 50.8; H, 3.49; N, 10.77; S, 12.33. Found: C, 50.45; H, 3.47; N, 10.62; S, 12.48. MS: [M + H]^+^ = 260.9 (exact mass 260.04).

***N*-(1-hydroxy-1,3-dihydrobenzo[*c*][1,2]oxaborol-6-yl)quinoxaline-2-carboxamide (17).** Yellow solid. Yield: 19%. mp 231.4–233.0 °C. ^1^H NMR (500 MHz, dmso) δ 10.86 (s, 1H, CONH), 9.55 (s, 1H, QxH), 9.28 (s, 1H, OH), 8.33 (d, *J* = 2.1 Hz, 1H, ArH), 8.29 (m, 1H, QxH), 8.25–8.18 (m, 1H, QxH), 8.01 (m, 2H, QxH), 7.91 (dd, *J* = 8.2, 2.1 Hz, 1H, ArH), 7.43 (d, *J* = 8.2 Hz, 1H, ArH), 4.99 (s, 2H, CH_2_). ^13^C NMR (126 MHz, dmso) δ 162.19, 150.03, 145.01, 144.17, 143.12, 139.87, 137.15, 132.27, 131.57, 129.76, 129.32, 124.01, 122.70, 121.83, 69.98. ^11^B NMR (160 MHz, dmso) δ 31.35. IR (ATR-Ge, cm^−1^): 3376 (ν CONH), 3320 (ν O-H), 2965 (ν CH_2_), 1680 (δ, C=O), 1539 (δ CONH), 1370 (ν B-O), 983 (ν C-O ether). Analysis calculated for C_16_H_12_BN_3_O_3_ (M_r_ 305.1): C, 62.99; H, 3.96; N, 13.77. Found: C, 63.46; H, 3.79; N, 13.51. MS: [M + H]^+^ = 306.0 (exact mass 305.10).

***N*-(1-hydroxy-1,3-dihydrobenzo[*c*][1,2]oxaborol-6-yl)-3-oxo-3,4-dihydroquinoxaline-2-carboxamide (18).** Yellow solid. Yield: 71%. mp 348.9–350.2 °C (decomp.). ^1^H NMR (600 MHz, dmso) δ 12.92 (s, 1H, LmH), 11.10 (s, 1H, CONH), 9.28 (s, 1H, OH), 8.12 (d, *J* = 2.1 Hz, 1H, ArH), 7.92–7.87 (m, 1H, ArH), 7.75 (dd, *J* = 8.2, 2.1 Hz, 1H, ArH), 7.68–7.62 (m, 1H, ArH), 7.44–7.37 (m, 3H, ArH), 4.97 (s, 2H, CH_2_). ^13^C NMR (151 MHz, dmso) δ 162.06, 154.43, 152.31, 150.11, 137.75, 133.05, 132.52, 131.77, 129.86, 124.58, 123.08, 122.44, 121.90, 116.24, 70.29. ^11^B NMR (160 MHz, dmso) δ 31.28. IR (ATR-Ge, cm^−1^): 3328 (ν O-H), 1706 (δ, C=O), 1550 (δ CONH), 1369 (ν B-O), 973 (ν C-O ether). Analysis calculated for C_16_H_12_BN_3_O_4_ (M_r_ 321.1): C, 59.85; H, 3.77; N, 13.09. Found: C, 59.38; H, 3.68; N, 12.73. MS: [M + H]^+^ = 322.0 (exact mass 321.09).

***N*-(1-hydroxy-1,3-dihydrobenzo[*c*][1,2]oxaborol-6-yl)quinoline-2-carboxamide (19).** Light yellow solid. Yield: 87%. mp 201.0–203.1 °C. ^1^H NMR (600 MHz, dmso) δ 10.76 (s, 1H, CONH), 9.26 (brs, 1H, OH), 8.62 (dd, *J* = 8.6, 0.8 Hz, 1H, ArH), 8.34 (d, *J* = 2.0 Hz, 1H, ArH), 8.25 (d, *J* = 8.5 Hz, 2H, ArH), 8.11 (dd, *J* = 8.2, 1.4 Hz, 1H, ArH), 7.95–7.88 (m, 2H, ArH), 7.75 (ddd, *J* = 8.1, 6.8, 1.2 Hz, 1H, ArH), 7.43 (dd, *J* = 8.1, 0.8 Hz, 1H, ArH), 4.99 (s, 2H, CH_2_). ^13^C NMR (151 MHz, dmso) δ 163.21, 150.69, 150.10, 146.45, 138.74, 137.65, 131.60, 131.21, 129.89, 129.47, 128.88, 128.68, 124.08, 122.69, 122.17, 119.30, 70.33. ^11^B NMR (160 MHz, dmso) δ 32.53. IR (ATR-Ge, cm^−1^): 3355 (ν O-H), 3319 (ν CONH), 1675 (δ, C=O), 1528 (δ CONH), 1361 (ν B-O), 976 (ν C-O ether). Analysis calculated for C_17_H_13_BN_2_O_3_ (M_r_ 304.11): C, 67.14; H, 4.31; N, 9.21. Found: C, 66.88; H, 4.04; N, 9.18. MS: [M + H]^+^ = 305.0 (exact mass 304.10).

### 3.3. Biological Studies

#### 3.3.1. Antimicrobial Screening

Antimycobacterial Screening against Mtb H37Ra, *M. smegmatis*, *M. aurum*, *M. avium*, and *M. kansasii*

The antimycobacterial assay was performed using fast-growing *Mycolicibacterium smegmatis* DSM 43,465 (ATCC 607), *Mycolicibacterium aurum* DSM 43,999 (ATCC 23366); non-tuberculous (atypical) mycobacteria, namely *Mycobacterium avium* DSM 44,156 (ATCC 25291) and *Mycobacterium kansasii* DSM 44,162 (ATCC 12478), purchased from German Collection of Microorganisms and Cell Cultures (Braunschweig, Germany); and an avirulent strain of *Mycobacterium tuberculosis* H37Ra ITM-M006710 (ATCC 9431) purchased from Belgian Co-ordinated Collections of Microorganisms (Antwerp, Belgium). The technique used for activity determination was the microdilution broth panel method using 96-well microtitration plates [32]. The culture medium was Middlebrook 7H9 broth (Merck, Darmstadt, Germany) enriched with glycerol (Merck, Darmstadt, Germany) and Middlebrook OADC growth supplement (Himedia, Mumbai, India) according to manufacturer instructions. 

The mycobacterial strains were cultured on supplemented Middlebrook 7H9 agar and suspensions were prepared in supplemented Middlebrook 7H9 broth. The final density was adjusted to value 1.0 according to the McFarland scale and diluted in the ratio 1:20 (for fast-growing mycobacteria) or 1:10 (for the rest of mycobacteria) with broth. 

The tested compounds were dissolved in DMSO (Sigma-Aldrich, St. Louis, MO, USA), then Middlebrook 7H9 broth was added to obtain a concentration of 2000 μg/mL. The standards used for activity determination were isoniazid (INH), rifampicin (RIF), and ciprofloxacin (CIP) (Merck). The final concentrations were reached using binary dilution and the addition of mycobacterial suspension and were set as 500, 250, 125, 62.5, 31.25, 15.625, 7.81, and 3.91 μg/mL. The final concentration of DMSO in any well did not exceed 2.5% (*v*/*v*) and did not affect the growth of mycobacteria. Positive (broth, DMSO, bacteria) and negative (broth, DMSO) growth controls were included.

The plates with slow-growing mycobacteria were sealed with polyester adhesive film and all the plates were incubated in the dark at 37 °C without agitation. The addition of 0.01% solution of resazurin sodium salt followed after 48 h of incubation for *M. smegmatis*, after 72 h of incubation for *M. aurum,* after 96 h of incubation for *M. avium* and *M. kansasii*, and after 120 h of incubation for *Mtb* H37Ra, respectively. The microtitration panels were then incubated for a further 2.5 h for the determination of activity against *M. smegmatis*, 4 h for *M. aurum*, 5–6 h for *M. avium* and *M. kansasii*, and 18 h for *Mtb* H37Ra, respectively. The antimycobacterial activity was expressed as the minimum inhibitory concentration (MIC). The MIC (in μg/mL) was determined on the basis of stain color change (blue color—active; pink color—not active). All the experiments were conducted in duplicates.

##### Antimycobacterial Screening against Mtb H37Rv and MDR Strains of Mtb

The microdilution method based on Microplate Alamar Blue Assay (MABA) was applied [32]. The tested strain *Mtb* H37Rv CNCTC My 331/88 (ATCC 27294) was obtained from the Czech National Collection of Type Cultures (CNCTC), National Institute of Public Health (Prague, Czech Republic). Multi-drug resistant strains of *Mtb* laboratory ID designation IZAK and MATI were obtained from the Department of Clinical Microbiology, University Hospital Hradec Králové from Dr. Pavla Paterová. The Middlebrook 7H9 broth of declared pH 6.6 (Sigma-Aldrich, St. Louis, MO, USA) enriched with 0.4% of glycerol (Sigma-Aldrich, St. Louis, MO, USA) and 10% of OADC growth supplement (Himedia, Mumbai, India) was used for cultivation. 

Tested compounds were dissolved and diluted in DMSO and mixed with broth (25 μL of DMSO solution in 2.475 mL of broth) and placed (100 μL) into microplate wells. The mycobacterial inocula were suspended in isotonic saline solution and the density was adjusted to 0.5–1.0 according to McFarland scale. These suspensions were diluted by 10^−1^ and used to inoculate the testing wells, adding 100 μL of mycobacterial suspension per well. The final concentrations of tested compounds in wells were 100, 50, 25, 12.5, 6.25, 3.13, and 1.56 μg/mL. INH was used as the standard (inhibition of growth). The positive control (visible growth) consisted of broth plus mycobacterial suspension plus DMSO. A total of 30 μL of Alamar Blue working solution (1:1 mixture of 0.02% resazurin sodium salt (aq. sol.) and 10% Tween 80) was added after five days of incubation. The results were then determined after 24 h of incubation. The MIC (in μg/mL) was determined as the lowest concentration that prevented the from blue to pink color change. All the experiments were conducted in duplicates.

##### Susceptibility Profiles for the Used MDR Mtb Strains

*Mtb* laboratory ID IZAK, isolated from a 63-year-old man from bronchial aspirate in 2020, was tested and interpreted according to CLSI (Clinical and Laboratory Standards Institute) breakpoints in 2020. 

*Mtb* laboratory ID MATI, isolated from a 23-year-old man from sputum in 2021, was tested and interpreted according to CLSI breakpoints in 2021. The drug susceptibility is summarized in Table 4.

#### 3.3.2. Cytotoxicity Screening

The human hepatocellular liver carcinoma cell line HepG2 purchased from Health Protection Agency Culture Collections (ECACC, Salisbury, UK) was cultured in DMEM (Dulbecco’s Modified Eagle’s Medium–high glucose) (Sigma-Aldrich, St. Louis, MO, USA) supplemented with 10% fetal bovine serum (PAA Laboratories GmbH, Pasching, Austria), 1% L-glutamine solution (Sigma-Aldrich, St. Louis, MO, USA), and non-essential amino acid solution (Sigma-Aldrich, St. Louis, MO, USA) in a humidified atmosphere containing 5% CO_2_ at 37 °C. For subculturing, the cells were harvested after trypsin/EDTA (Sigma-Aldrich, St. Louis, MO, USA) treatment at 37 °C. For cytotoxicity evaluation, the cells treated with the tested substances were used as experimental groups. Untreated HepG2 cells served as controls.

The cells were seeded in a density of 10,000 cells per well in a 96-well plate 24 h prior to the experiment. The next day, the cells were treated with each of the tested substances dissolved in DMSO. The tested substances were prepared at different incubation concentrations (1–1000 μM) in triplicates according to their solubility. Concurrently, the controls representing 100% cell viability, 0% cell viability (the cells treated with 10% DMSO), no cell control, and vehiculum controls were prepared in triplicates. After 24 h of incubation in a humidified atmosphere containing 5% CO_2_ at 37 °C, the reagent from the kit CellTiter 96 AQueous One Solution Cell Proliferation Assay (CellTiter 96, PROMEGA, Fitchburg, WI, USA) was added. After 2 h of incubation at 37 °C, the absorbance of the samples was recorded at 490 nm (TECAN, Infinita M200, Grödig, Austria). A standard toxicological parameter IC_50_ was calculated using nonlinear regression from a semilogarithmic plot of incubation concentration versus the percentage of absorbance (log(inhibitor) vs. normalized response model, least squares fit) relative to untreated controls using GraphPad Prism 9 software, (GraphPad Software, San Diego, CA, USA).

## 4. Conclusions

Within this work, nineteen derivatives of *N*-(1-hydroxy-1,3-dihydrobenzo[*c*][1,2]oxaborol-6-yl)(hetero)aryl-2-carboxamides have been prepared and fully characterized. In the in vitro biological antimicrobial screening, the compounds showed selective growth inhibition of the tested mycobacteria in comparison to no growth inhibition of the representatives of Gram-positive and Gram-negative bacteria, yeasts, and fungi. The selected compounds retained the biological activity also against resistant strains of clinical isolates of *Mtb* H37Rv. The majority of the most active compounds proved to possess a beneficial selectivity index concerning the negligible inhibition of the HepG2 cell line. This series of compounds provides a point for further research of new antimycobacterial compounds.

## Figures and Tables

**Table 1:** Calculated lipophilicity of the prepared compounds (log*P*), comparison of their antimycobacterial activity (MIC) with standards, and their cytotoxicity against HepG2 (IC_50_).

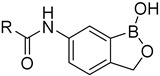									
		Log*P*	MIC						IC_50_
Comp.	R		*Mtb* H37Ra (µM)	*Mtb* H37Rv (µM)	*M. kansasii* (µM)	*M. avium* (µM)	*M. aurum* (µM)	*M. smeg*. (µM)	HepG2 (µM)
**1**	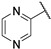	0.42	122.53	49.01	30.62	122.53	15.33	30.62	>1000
**2**	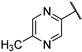	0.92	58.07	46.46	58.07	58.07	14.53	29.03	>500
**3**	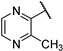	0.58	>1851.4	92.91	58.07	>1851.4	14.53	14.53	>500
**4**	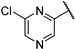	1.16	107.95	86.36	**26.98**	**26.98**	13.51	26.98	>1000
**5**	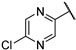	1.16	**26.98**	**21.59**	107.95	53.98	13.51	53.98	82.44
**6**	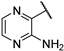	0.67	>1851.47	>370.29	>1851.47	>1851.47	14.48	462.87	>100
**7**	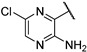	1.40	>1851.47	>328.41	>1851.47	410.51	>1851.47	410.51	>100
**8**	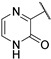	−0.61	230.59	92.24	230.59	57.65	28.82	28.82	>1000
**9**	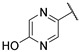	1.05	115.30	184.48	115.30	230.59	230.59	57.65	>1000
**10**	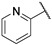	1.20	61.50	49.20	30.74	61.50	15.39	61.50	>1000
**11**	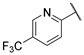	2.19	97.03	**9.72**	**12.14**	97.03	24.25	24.25	>100
**12**	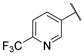	1.84	194.07	155.26	194.07	388.14	**12.14**	24.25	>250
**13**	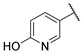	1.19	462.88	>370.30	115.72	462.88	115.72	115.72	>1000
**14**	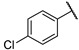	2.52	434.77	43.48	217.39	434.77	13.60	217.39	>1000
**15**	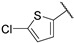	2.27	53.23	42.59	106.46	425.85	53.23	851.71	210.5
**16**	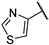	1.06	120.16	96.13	30.03	240.32	15.03	30.03	>1000
**17**	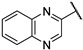	2.01	>819.40	**20.49**	**25.60**	102.43	**12.82**	**12.82**	>250
**18**	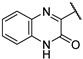	0.98	>1851.47	>311.43	>1851.47	48.66	24.32	24.32	243.7
**19**	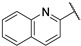	2.59	205.52	82.21	205.52	**25.68**	25.68	25.68	466.7
	INH	-	3.65	2.84	45.57	7291.87	28.51	227.87	na
	RIF	-	0.00759	na	0.06	0.08	0.47	15.19	na
	CIP	-	1.51	na	0.75	1.51	0.05	0.19	na
	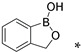		na	55.99 *	na	na	na	na	na
	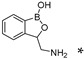		na	11.04 *	na	na	na	na	na

Used standards: INH = isoniazid, RIF = rifampicin, CIP = ciprofloxacin; * compounds synthesized and evaluated by Palencia et al. [13]; na = not available.

**Table 2:** Comparison of the antimycobacterial activity of selected compounds against collection strain of *Mtb* H37Rv and the clinical isolates of MDR strains of *Mtb* (labeled IZAK and MATI) including standards.

Comp.	MIC *Mtb* H37Rv (µM)	MIC *Mtb* IZAK (µM)	MIC *Mtb* MATI (µM)
**4**	86.36	86.36	86.36
**5**	21.59	43.18	43.18
**10**	49.20	98.41	98.41
**17**	20.49	40.97	40.97
INH	2.84	91.15	>91.15
CPX	0.6	0.6	0.6
EMB	1.91	7.64	7.64

**Table 3:** Cytotoxicity of the selected compounds against HepG2 cell line (IC_50_) and selectivity indices (SI) in relation to their antimycobacterial activity against *Mtb* H37Rv (MIC).

Compound	HepG2 IC_50_ (µM)	*Mtb* H37Rv MIC (µM)	SI
**1**	>1000	49.01	>20.40
**2**	>500	46.46	>10.76
**5**	82.44	21.59	3.82
**10**	>1000	49.20	>20.33
**11**	>100	9.72	>10.29
**14**	>1000	43.48	>23.00
**15**	210.5	42.59	4.94
**17**	>250	20.49	>12.20

**Table 4:** Susceptibility profiles of tested MDR *Mtb* strains.

Laboratory ID	Drug	Concentration (µM)	Susceptibility
IZAK	STM	6.88	resistant
	INH	29.17	resistant
	RIF	>9.72	resistant
	EMB	2.45	sensitive
	PZA	>129.96	resistant
MATI	STM	>27.51	resistant
	INH	>58.33	resistant
	RIF	>9.72	resistant
	EMB	2.45	sensitive
	PZA	>1039.70	resistant

STM = streptomycin; INH = isoniazid; RIF = rifampicin; EMB = ethambutol; PZA = pyrazinamide.

## Data Availability

Data are contained within the article or Supplementary Materials. All original data presented in this study are available from the authors upon request.

## Supplementary Materials

The following supporting information can be downloaded at: https://www.mdpi.com/article/10.3390/ijms24032951/s1.
