# Supplementary material for: Design, Synthesis and Antimicrobial Evaluation of New N-(1-Hydroxy-1,3-dihydrobenzo[c][1,2]oxaborol-6-yl)(hetero)aryl-2-carboxamides as Potential Inhibitors of Mycobacterial Leucyl-tRNA Synthetase
Source: Int J Mol Sci. 2023 Feb 2;24(3):2951. doi: 10.3390/ijms24032951 (PMC9917560; doi:10.3390/ijms24032951)
Supplement: Supplementary file 1 [file ijms-24-02951-s001.zip › ijms-2166579-supplementary.pdf]

Design, Synthesis and Antimicrobial Evaluation of New *N*-(1-hydroxy-1,3-dihydrobenzo[c][1,2]oxaborol-6-yl)(hetero)aryl-2-carboxamides as Potential Inhibitors of Mycobacterial Leucyl-tRNA Synthetase

Petr Šlechta[1], Adam Anthony Needle[1], Ondřej Janďourek[2], Pavla Paterová[3], Klára Konečná[2], Pavel Bárta[4], Jiří Kuneš[5], Vladimír Kubíček[4], Martin Doležal[1], and Marta Kučerová-Chlupáčová[1]*

[1] Department of Pharmaceutical Chemistry and Pharmaceutical Analysis, Faculty of Pharmacy in Hradec Králové, Charles University, 500 05 Hradec Králové, Czech Republic

[2] Department of Biological and Medical Sciences, Faculty of Pharmacy in Hradec Králové, Charles University, 500 05 Hradec Králové, Czech Republic

[3] Department of Clinical Microbiology, University Hospital, 500 05 Hradec Králové, Czech Republic

[4] Department of Biophysics and Physical Chemistry, Faculty of Pharmacy in Hradec Králové, Charles University, 500 05 Hradec Králové, Czech Republic

[5] Department of Organic and Bioorganic Chemistry, Faculty of Pharmacy in Hradec Králové, Charles University, 500 05 Hradec Králové, Czech Republic

* Corresponding author: M.K.-C.: kucerom@faf.cuni.cz, https://sciprofiles.com/profile/130526

Supplementary Materials

1. Materials and Methods

1.1. Antibacterial Activity Screening

The microdilution broth method was performed according to EUCAST (The European Committee on Antimicrobial Susceptibility Testing) instructions [1] with slight modifications. Eight tested bacterial strains (four G+ and four G-) were purchased from the Czech Collection of Microorganisms (CCM, Brno, Czech Republic) or the German Collection of Microorganisms and Cell Cultures (DSM, Braunschweig, Germany): *Staphylococcus aureus* subsp. *aureus* CCM 4223 (ATCC 29213), methicillin-resistant *Staphylococcus aureus* subsp. *aureus* (MRSA) CCM 4750 (ATCC 43300), *Staphylococcus epidermidis* CCM 4418 (ATCC 12228), *Enterococcus faecalis* CCM 4224 (ATCC 29212), *Escherichia coli* CCM 3954 (ATCC 25922), *Klebsiella pneumoniae* CCM 4415 (ATCC 10031), *Acinetobacter baumannii* DSM 30007 (ATCC 19606), *Pseudomonas aeruginosa* CCM 3955 (ATCC 27853). The cultivation was done in Cation-adjusted Mueller-Hinton broth (CAMHB, M-H 2 Broth, Merck, Darmstadt, Germany) at 35 ± 2 °C.

Tested compounds were dissolved in DMSO (Merck) to produce stock solutions. The final concentration of DMSO in the cultivation medium did not exceed 1% (v/v) of the total solution composition and did not affect the growth of bacteria. Positive growth controls consisted of test microbe solely, while negative growth controls consisted of cultivation medium and DMSO. Antibacterial activity was expressed as minimum inhibitory concentration (MIC, in µM) after 24 and 48 h of static incubation in the dark and humidified atmosphere, at 35 ± 2 °C. Visual inspection and metabolic activity indicator, Alamar Blue (AlamarBlueTM Cell Viability reagent, ThermoFisher Scientific, USA), were used for MIC endpoint evaluation. The internal quality standards of gentamicin and ciprofloxacin (both from Merck) were involved in assays (for MIC of standards, see below).

1.2. Antifungal Activity Screening

Antifungal activity evaluation was performed using a microdilution broth method according to EUCAST instructions [2,3] with slight modifications. Eight fungal strains (four yeasts and four molds) were used for antifungal activity screening, namely: *Candida albicans* CCM 8320 (ATCC 24433), *Candida krusei* CCM 8271 (ATCC 6258), *Candida parapsilosis* CCM 8260 (ATCC 22019), *Candida tropicalis* CCM 8264 (ATCC 750), *Aspergillus fumigatus* ATCC 204305, *Aspergillus flavus* CCM 8363, *Lichtheimia corymbifera* CCM 8077, and *Trichophyton interdigitale* CCM 8377 (ATCC 9533). Tested

strains were purchased from the Czech Collection of Microorganisms (CCM, Brno, Czech Republic) or the American Type Collection Cultures (ATCC, Manassas, VA, USA).

Tested compounds were dissolved in DMSO and diluted in a two-fold manner with RPMI 1640 medium, glutamine, and 2% glucose, buffered to pH 7.0 with MOPS (3-morpholinopropane-1-sulfonic acid). The final concentration of DMSO in the testing medium did not exceed 1% (v/v) of the total solution composition. Static incubation was performed in the dark and a humid atmosphere, at 35 ± 2 °C, for 24 and 48 h (72 and 120 h for *Trichophyton interdigitale*, respectively). Positive growth controls consisted of test microbe solely, while negative growth controls consisted of cultivation medium and DMSO. Visual inspection and metabolic activity indicator, Alamar Blue (ThermoFisher Scientific, USA), were used for MIC endpoint evaluation. The internal quality standards, amphotericin B (Merck), and voriconazole (Toronto Research Chemicals, CA) were involved in assays (for IC_{50}, IC_{90}, and MIC of standards, see below).

2. Results

2.1. *In Silico* Study

Table S1: Score values from the template docking of the adducts of the synthesized compound and AMP into mtbLeuRS (PDB ID: 5AGR).

Comp.	S (Score)
**11**	-10.318074
**17**	-10.229079
**7**	-10.112683
**10**	-10.103503
**4**	-10.060172
**2**	-10.010665
**9**	-9.9291506
**8**	-9.4784718
**1**	-9.4719524
**18**	-9.3661909
**12**	-9.2085085
**19**	-8.8039217
**14**	-8.739666
**6**	-8.701005
**3**	-8.6372814
**13**	-8.5417795
**5**	-8.4964485
**15**	-8.4129076
**16**	-8.267931

2.2. Antibacterial Activity Screening

*Table S2: Antibacterial activity of compounds **1–19** against tested bacterial species.*

**Strain**	**Compound–MIC** (μM)																			
	**Incb.**	**1**	**2**	**3**	**4**	**5**	**6**	**7**	**8**	**9**	**10**	**11**	**12**	**13**	**14**	**15**	**16**	**17**	**18**	**19**
Staphylococcus aureus subsp. *aureus*	24h	**250**	**250**	>500	**125**	>500	>500	>125	**125**	>500	**250**	>125	>500	>500	>500	**500**	**125**	>500	>500	**125**
	48h	**250**	**250**	>500	**125**	>500	>500	>125	**125**	>500	**250**	>125	>500	>500	>500	**500**	**125**	>500	>500	**125**
Methicillin-resistant *Staphylococcus aureus subsp. aureus*	24h	**500**	>500	>500	**250**	>500	>500	>125	**125**	>500	**250**	>125	>500	>500	>500	**500**	**250**	>500	>500	>125
	48h	**500**	>500	>500	**250**	>500	>500	>125	**125**	>500	**250**	>125	>500	>500	>500	**500**	**500**	>500	>500	>125
Staphylococcus epidermidis	24h	**500**	**500**	>500	**500**	>500	>500	>125	**500**	>500	**500**	>125	>500	>500	>500	**500**	**500**	>500	>500	>125
	48h	**500**	**500**	>500	**500**	>500	>500	>125	**500**	>500	**500**	>125	>500	>500	>500	**500**	>500	>500	>500	>125
Enterococcus faecalis	24h	>500	>500	>500	>500	>500	>500	>125	>500	>500	>500	>125	>500	>500	>500	>500	>500	>500	>500	>125
	48h	>500	>500	>500	>500	>500	>500	>125	>500	>500	>500	>125	>500	>500	>500	>500	>500	>500	>500	>125
Escherichia coli	24h	>500	>500	>500	>500	>500	>500	>125	>500	>500	>500	>125	>500	>500	>500	>500	**500**	>500	>500	>125
	48h	>500	>500	>500	>500	>500	>500	>125	>500	>500	>500	>125	>500	>500	>500	>500	>500	>500	>500	>125
Klebsiella pneumoniae	24h	>500	>500	>500	>500	>500	>500	>125	>500	>500	>500	>125	>500	>500	>500	>500	**500**	>500	>500	>125
	48h	>500	>500	>500	>500	>500	>500	>125	>500	>500	>500	>125	>500	>500	>500	>500	>500	>500	>500	>125
Acinetobacter baumannii	24h	>500	>500	>500	>500	>500	>500	>125	>500	>500	>500	>125	>500	>500	>500	>500	>500	>500	>500	>125
	48h	>500	>500	>500	>500	>500	>500	>125	>500	>500	>500	>125	>500	>500	>500	>500	>500	>500	>500	>125
Pseudomonas aeruginosa	24h	>500	>500	>500	>500	>500	>500	>125	>500	>500	>500	>125	>500	>500	>500	>500	>500	>500	>500	>125
	48h	>500	>500	>500	>500	>500	>500	>125	>500	>500	>500	>125	>500	>500	>500	>500	>500	>500	>500	>125

Note: MIC – minimum inhibitory concentration.

Table S3: Results of internal quality controls (standards) in antibacterial activity screening.

Internal quality control/standard	ciprofloxacin (µM)		gentamicin (µM)	
Bacterial strain	MIC (spectrophotometric detection, 530 nm*)	MIC (visual detection**)	MIC (spectrophotometric detection, 530 nm*)	MIC (visual detection**)
Staphylococcus aureus spp. *aureus* CCM 4223	0.773	0.386—0.773	2.094	1.047
Staphylococcus aureus spp. *aureus* MRSA, CCM 4750	0.386	0.386	>16.751	>16.751
Staphylococcus epidermidis, CCM 4418	0.773	0.386	0.131	0.131
Enterococcus faecalis, CCM 4224	3.090	3.090	>16.751	>16.751
Escherichia coli, CCM 3954	0.024	0.024	2.094	>2.094
Klebsiella pneumoniae, CCM 4415	0.193	0.386	1.047	1.047
Acinetobacter baumannii, DSM 30007	1.545	1.545	16.751	16.751
Pseudomonas aeruginosa, CCM 3955	1.545	0.773	1.047	1.047

Notes: Spectrophotometric detection – results were read with a microdilution plate reader (SynergyTM HTX, BioTek Instruments, Inc., USA) at wavelength 530 nm. MIC – minimum inhibitory concentration

*The MIC of antibacterial agents is the lowest concentration giving rise to an inhibition of growth of 95% of that of the drug-free control. Results were read 24 h after incubation without agitation at 35±2°C in a humidified atmosphere.

**The MIC was determined by the naked eye in the well with the lowest drug concentration, where no visible growth of microbial agent was detected. Results were read after 24 h incubation without agitation at 35±2°C in a humidified atmosphere.

2.3. Antifungal Activity Screening

*Table S4: Antifungal activity of compounds **1–19** against tested fungal species.*

Strain	Compound–MIC (μM)																			
	**Incb.**	**1**	**2**	**3**	**4**	**5**	**6**	**7**	**8**	**9**	**10**	**11**	**12**	**13**	**14**	**15**	**16**	**17**	**18**	**19**
Candida albicans	24h	>500	>500	>500	>500	>500	>500	>125	>500	>500	>500	>125	>500	>500	>125	>500	>500	>500	>125	>125
	48h	>500	>500	>500	>500	>500	>500	>125	>500	>500	>500	>125	>500	>500	>125	>500	>500	>500	>125	>125
Candida krusei	24h	>500	>500	>500	>500	>500	>500	>125	>500	>500	>500	>125	>500	>500	>125	>500	>500	>500	>125	>125
	48h	>500	>500	>500	>500	>500	>500	>125	>500	>500	>500	>125	>500	>500	>125	>500	>500	>500	>125	>125
Candida parapsilosis	24h	>500	>500	>500	>500	>500	>500	>125	>500	>500	>500	>125	>500	>500	>125	>500	>500	>500	>125	>125
	48h	>500	>500	>500	>500	>500	>500	>125	>500	>500	>500	>125	>500	>500	>125	>500	>500	>500	>125	>125
Candida tropicalis	24h	>500	>500	>500	>500	>500	>500	>125	>500	>500	>500	>125	>500	>500	>125	>500	>500	>500	>125	>125
	48h	>500	>500	>500	>500	>500	>500	>125	>500	>500	>500	>125	>500	>500	>125	>500	>500	>500	>125	>125
Aspergillus fumigatus	24h	>500	>500	>500	>500	>500	>500	>125	>500	>500	>500	>125	>500	>500	>125	>500	>500	>500	>125	>125
	48h	>500	>500	>500	>500	>500	>500	>125	>500	>500	>500	>125	>500	>500	>125	>500	>500	>500	>125	>125
Aspergillus flavus	24h	>500	>500	>500	>500	>500	>500	>125	>500	>500	>500	>125	>500	>500	>125	>500	>500	>500	>125	>125
	48h	>500	>500	>500	>500	>500	>500	>125	>500	>500	>500	>125	>500	>500	>125	>500	>500	>500	>125	>125
Lichtheimia corymbifera	24h	>500	>500	>500	>500	>500	>500	>125	>500	>500	>500	>125	>500	>500	>125	>500	>500	>500	>125	>125
	48h	>500	>500	>500	>500	>500	>500	>125	>500	>500	>500	>125	>500	>500	>125	>500	>500	>500	>125	>125
Trichophyton interdigitale	72h	>500	>500	>500	>500	>500	>500	>125	>500	>500	>500	>125	>500	>500	>125	>500	>500	>500	>125	>125
	120h	>500	>500	>500	>500	>500	>500	>125	>500	>500	>500	>125	>500	>500	>125	>500	>500	>500	>125	>125

Note: MIC - minimum inhibitory concentration.

Table S5: Results of internal quality controls (standards) in antifungal screening.

Internal quality control/standard	amphotericin B (μM)		voriconazole (μM)	
Yeast/mold strain	IC_{90} (spectrophotometric detection, 530 nm**)	MIC (visual detection*)	IC_{50} (spectrophotometric detection, 530 nm***)	visual detection*
Candida albicans, CCM 8320	1.082	1.082	0.086	>45.806
Candida krusei, CCM 8271	1.082	1.082	0.716	>45.806
Candida parapsilosis, CCM 8260	0.541	0.541	0.086	>45.806
Candida tropicalis, CCM 8321	1.082	1.082	0.179	>45.806
Aspergillus fumigatus, ATCC 204305	4.329	4.329	0.716	2.863
Aspergillus flavus, CCM 8363	4.329	4.329	5.726	>45.806
Lichtheimia corymbifera CCM 8077	1.082	1.082	>45.806	>45.806
Trichophyton interdigitale, CCM 8377	1.082	1.082	2.863	>45.806

Notes: Spectrophotometric detection- results were read with a microdilution plate reader (SynergyTM HTX, BioTek Instruments, Inc., USA) at wavelength 530 nm. MIC – minimum inhibitory concentration

*The MIC was determined by the naked eye in the well with the lowest drug concentration, where no visible growth of antifungal agent was detected. Results were read after 24 h (bacteria, yeasts) or 48 h (molds) microdilution plates cultivation without agitation at 35±2°C in a humidified atmosphere.

**The IC_{90} of amphotericin B is the lowest concentration giving rise to an inhibition of growth of 90% of that of the drug-free control. Results were read after 24 h (yeasts) or 48 h (molds) microdilution plates cultivation without agitation at t 35±2°C in a humidified atmosphere.

***The IC_{50} of voriconazole is the lowest drug concentration giving inhibition of growth of 50% of that of the drug-free control. Results were read after 24 h (yeasts) or 48 h (molds) microdilution plates cultivation without agitation at t 35±2°C in a humidified atmosphere.

References:

1. European Committee for Antimicrobial Susceptibility Testing (EUCAST) of the European Society for Clinical Microbiology and Infectious Diseases (ESCMID). Eucast Discussion Document E. Dis 5.1: Determination of minimum inhibitory concentrations (MICs) of antibacterial agents by broth dilution. *Clin. Microbiol. Infect.* **2003**, *9*, 9-15, doi:10.1046/j.1469-0691.2003.00790.x.
2. Arendrup, M.C.; Meletiadis, J.; Mouton, J.W.; Lagrou, K.; Hama, P.; Guinea, J.; AFST-EUCAST. EUCAST DEFINITIVE DOCUMENT E.DEF 7.3.1. Method for the Determination of Broth Dilution Minimum Inhibitory Concentrations of Antifungal Agents for Yeasts. **2017**, 1-21, doi:https://www.eucast.org/fileadmin/src/media/PDFs/EUCAST_files/AFST/Files/EUCAST_E_Def_7_3_1_Yeast_testing__definitive.pdf.
3. Arendrup, M.C.; Meletiadis, J.; Mouton, J.W.; Lagrou, K.; Hamal, P.; Guinea, J.; AFST-EUCAST. EUCAST DEFINITIVE DOCUMENT E.DEF 9.3.1. Method for the Determination of Broth Dilution Minimum Inhibitory Concentrations of Antifungal Agents for Conidia Forming Moulds. **2017**, *EUCAST Definitive Document E.Def 9.3.1. 2017*, 1–23, doi:https://www.eucast.org/fileadmin/src/media/PDFs/EUCAST_files/AFST/Files/EUCAST_E_Def_9_3_1_Mould_testing__definitive.pdf
